# Development and usability testing of a depth camera–based web application for functionally relevant foot kinematics analysis

**DOI:** 10.3389/fmedt.2025.1677174

**Published:** 2025-10-03

**Authors:** Fitri Anestherita, Angela B. M. Tulaar, Maria Regina Rachmawati, Em Yunir, Dante Saksono Harbuwono, Retno Asti Werdhani, Ahmad Yanuar Safri, Muhammad Febrian Rachmadi, Muhammad Hanif Nadhif, Azwien Niezam Hawalie M, Boya Nugraha, Safa Nabila Putri, Fiska Fianita, Thasya Niken Saputri

**Affiliations:** ^1^ Doctoral Program in Medical Sciences, Faculty of Medicine, Universitas Indonesia, Dr. Cipto Mangunkusumo National General Hospital, Jakarta, Indonesia; ^2^ Department of Physical Medicine and Rehabilitation, Faculty of Medicine, Universitas Indonesia, Dr. Cipto Mangunkusumo National General Hospital, Jakarta, Indonesia; ^3^ Division of Endocrinology, Metabolism, and Diabetes, Department of Internal Medicine, Faculty of Medicine, Universitas Indonesia, Dr. Cipto Mangunkusumo National General Hospital, Jakarta, Indonesia; ^4^ Department of Community Medicine, Faculty of Medicine, Universitas Indonesia, Jakarta, Indonesia; ^5^ Department of Neurology, Faculty of Medicine, Universitas Indonesia, Dr. Cipto Mangunkusumo National General Hospital, Jakarta, Indonesia; ^6^ Faculty of Computer Science, Universitas Indonesia, Depok, Indonesia; ^7^ Medical Technology IMERI, Faculty of Medicine, Universitas Indonesia, Jakarta, Indonesia; ^8^ Department of Medical Physiology and Biophysics, Faculty of Medicine, Universitas Indonesia, Jakarta, Indonesia; ^9^ Department of Physical Medicine and Rehabilitation, Faculty of Medicine, Hannover Medical School, Hannover, Germany

**Keywords:** foot kinematics, web application, depth camera, gait analysis, clinical usability, musculoskeletal biomechanics

## Abstract

**Introduction:**

KineFeet, a depth camera-based web tool for analyzing functional foot kinematics, was developed and tested in this study. The program was optimized for usability, affordability, and clinical relevance through an iterative design and development process.

**Methods:**

The Azure Kinect DK camera records and analyzes sagittal and frontal plane foot movements in real time. A usability-focused study was created. Five physiatrists tested the KineFeet prototype for its ability to assess foot kinematics. Performance was measured by task completion success, error rate, and time. The System Usability Scale (SUS) measured user satisfaction. Quality assessments were also obtained through semi-structured interviews.

**Results:**

Participants achieved an average success rate of 96.29%, with an error rate of 0.074% and an average completion time of 10 min 11 s. Time-Based Efficiency (TBE) showed that user performance (0.0442 tasks/s) was 1.21 times slower than expert user performance (0.05348 tasks/s). SUS yielded an average score of 66.5, indicating a good level of satisfaction and user acceptance.

**Conclusion:**

KineFeet represents a promising innovation in assessing functional foot kinematics. The system demonstrated strong usability in preliminary testing and holds potential for broader clinical adoption following further development.

## 1 Introduction

Although static measures of foot posture are only weakly related to rearfoot or midfoot kinematics and have limited ability to predict dynamic movement, assessing foot kinematics is crucial for diagnosing and managing lower extremity musculoskeletal issues (1). Optical motion capture systems, widely recognized as the gold standard for gait analysis, face significant challenges due to their high cost, complex setup, and limited availability (2). Consequently, there is an increasing demand for more practical and clinically relevant alternatives. Developing affordable and easily accessible gait analysis tools via web-based software could democratize movement analysis and facilitate the creation of personalized treatment plans (3). Successfully building such a system requires a comprehensive understanding of biomechanics, software engineering, and data processing techniques (4).

The initial stage of developing a web platform involves selecting appropriate sensors and hardware components that are comprehensive and capable of capturing the necessary kinematic data with high fidelity. Depth camera technology, initially developed for gaming and consumer electronics, has demonstrated potential for motion tracking applications (5–7). Devices such as the Azure Kinect DK offer depth sensing and skeletal tracking capabilities at a markedly reduced price compared to advanced optical motion capture systems (8–11). A study using three depth sensors found that the Azure Kinect demonstrated better tracking performance for kinematic gait patterns during treadmill walking at non-frontal viewing angles compared to the Kinect v2 and Orbbec Astra Pro v2 sensors (12). Integrating such technology into a web-based platform has the potential to eliminate software installation requirements and enhance portability.

The KineFeet system was developed to overcome these challenges. KineFeet is a prototype web-based application that utilizes depth camera technology for clinical foot kinematic analysis. The main function of the application is to analyze the sagittal and frontal planes of the foot during gait, providing automatic detection of subphases in the stance phase of the gait cycle, real-time measurements, and data export options within a browser environment. This paper describes the design and development of KineFeet, presenting the results of a usability evaluation conducted with experienced clinicians. This study details the development process and assesses KineFeet's usability through both qualitative and quantitative methods. The usability testing involved clinicians to evaluate effectiveness, efficiency, and user satisfaction.

## 2 Materials and methods

This study was a software development and observational analytic research design to evaluate its usability. Data were collected at the Department of Physical Medicine and Rehabilitation, Dr. Cipto Mangunkusumo General National Hospital (Central Jakarta, Jakarta, Indonesia). The study protocol received approval and registration from the Research Ethics Committee of the Faculty of Medicine at the University of Indonesia (KET- 1586/UN2.F1/ETIK/PPM.00.02/2023). The research methodology was structured into several sequential phases, beginning with the iterative development of the KineFeet system, followed by technical validation and user-based usability testing.

### 2.1 Step 1: iterative system development

KineFeet was developed using a user-centered design methodology, incorporating feedback from clinical users throughout the development process. The system was constructed with the following goals: (1) Ensure ease of use for clinicians with minimal technical training. (2) Provide accurate sagittal plane angle measurements. (3) Enable real-time visualization and data storage. (4) Maximize accessibility through web-based deployment.

#### 2.1.1 Hardware components

##### 2.1.1.1 The computer

The system requires a computer equipped with a minimum of eight cores in the central processing unit (CPU), a graphics processing unit (GPU) with specifications equivalent to or stronger than the Nvidia GTX 1050, and at least 8 gigabytes (GB) of random access memory (RAM).

##### 2.1.1.2 Camera placement

**Figure 1:**
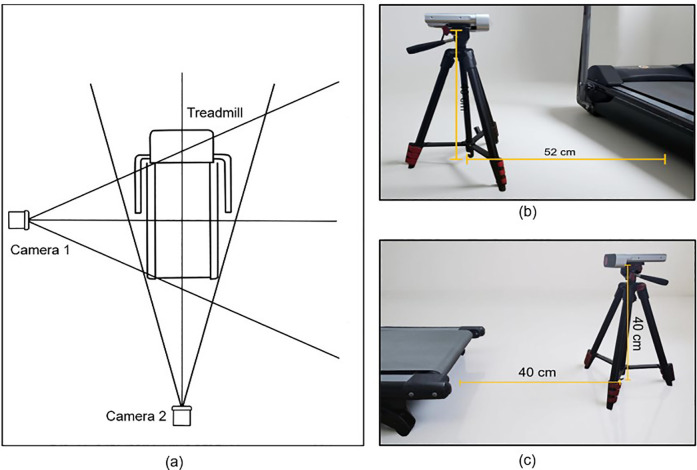
Camera placement. **(a)** Schematic placement of the cameras for KineFeet recording of the right foot, **(b)** Microsoft Azure Kinect Camera mounted on tripod at 40 cm height above the ground with a distance of 52 cm facing the left side of the treadmill, and **(c)** set the camera behind the treadmill with a distance of 40 cm.

KineFeet recorded simultaneously using two Microsoft Azure Kinect Cameras (Microsoft, Redmond, WA, USA) placed on the posterior and sides of the treadmill, showing the medial side of the foot. The camera was positioned at a distance that allowed the entire length of the treadmill belt to be visible, while still maintaining marker detection. The posterior camera was placed 40 cm behind the treadmill and the lateral camera 52 cm from the side of the treadmill. Both cameras were mounted on tripods with 40 cm height from the ground to the base of the camera (Figure 1).

##### 2.1.1.3 Marker

**Figure 2:**
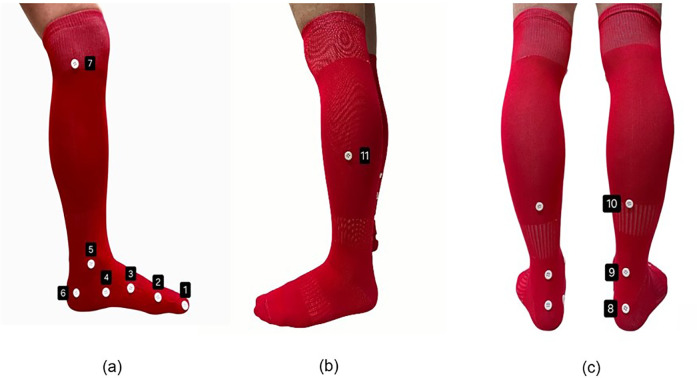
Placement of markers. **(a)** Medial side; (1) first distal interphalang joint, (2) head of first metatarsal, (3) in the middle of navicular tuberosity and head of first metatarsal on first metatarsal shaft, (4) navicular tuberosity, (5) medial malleolus, (6) calcaneal tu- berosity, (7) knee joint line, and **(b)** posterior side; (8) achilles tendon attachment, (9) on a line with medial malleolus, (10) gastrocnemius musculotendinous junction, and **(c)** on the lateral side of (11) half of a calf.

A white round button 1 cm in diameter is an effective marker to ensure good detection. Smaller sizes pose difficulty, while larger sizes are impractical due to the close distance between joints. Buttons as markers are ideal for Kinect Azure's depth sensor mechanism, capable of distinguishing objects from the background. Markers with 2 mm or greater are easily identified. Secure markers to socks with double-sided adhesive tape. Above-knee socks, with color contrast to buttons, provide uniform backgrounds, ensuring consistent detection of markers as angle's endpoints (Figure 2).

To further improve the reliability of marker detection, KineFeet's configuration was refined through an iterative process of testing and adjustment. High-contrast visibility between the marker and its background was prioritized; thus, white markers were consistently paired with red socks to enhance distinction. In order to reduce visual inconsistencies, all participants wore socks and markers made from identical materials and colors. Moreover, to prevent displacement caused by sock shifting or wrinkling during walking, snug-fitting socks were used and carefully positioned to ensure that markers remained securely in place throughout each trial.

##### 2.1.1.4 Workspace

**Figure 3:**
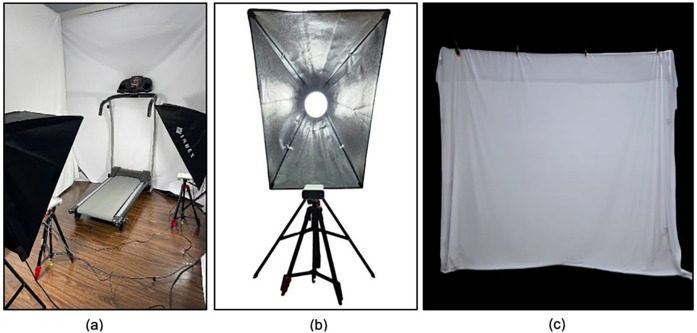
Workspace for kineFeet. **(a)** Gait analysis room set for Kinefeet recording, **(b)** placement of camera and studio light, and **(c)** white backdrop.

Ideal marker identification requires sufficient lighting. The best quality was obtained by placing a light source (3800 Lm light) behind each camera and utilizing a soft box to diffuse the light and minimize shadows on the backdrop screen. A white backdrop screen is placed to minimize distractions and improve lighting (Figure 3).

##### 2.1.1.5 Treadmill

Non-inclined treadmill or walking pad with a flat surface wide enough to allow a natural stride without the lower limbs crossing. A sufficient width has been shown to improve patient confidence and assist balance.

##### 2.1.1.6 Recording

The pattern of walking on a treadmill may differ from walking on land. Steps become shorter, and the attempt to maintain balance may result in altered lower limb kinematics. To reduce these variations, it is important to familiarize the patient with the mechanics of treadmill walking before recording. For patients with balance problems, the use of a body harness is recommended to increase confidence and reduce the risk of falls. Gradually increase the speed until patients are comfortable, aiming for a pace similar to their usual walking speed while avoiding holding onto the handlebars.

**Figure 4:**
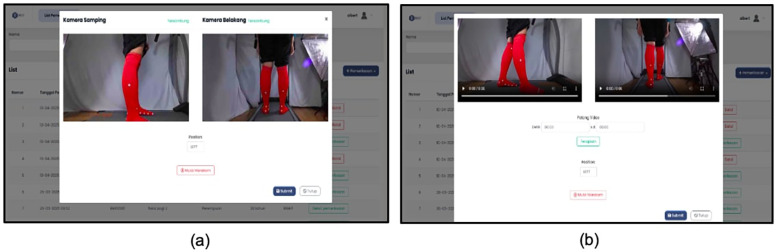
Recording kineFeet. **(a)** User interface to start recording after entering patient's data, and **(b)** user interface preparing to submit the video after completing the recording.

Video recordings were captured for a duration of 5 s using two Azure Kinect cameras positioned laterally and posteriorly. This 5-second window was strategically selected to capture a brief yet stable segment of the gait cycle, typically when the participant's walking pattern had stabilized and appeared most natural. The relatively short duration also facilitated faster data upload and processing through the KineFeet web application. Recording commenced once the participant's gait appeared steady (Figure 4).

#### 2.1.2 System architecture and data processing

The KineFeet system uses a cloud-based platform for automated gait analysis. Video is captured with Azure Kinect cameras and uploaded to a server for processing. A deep learning model (Convolutional Neural Network), trained on a substantial dataset of annotated anatomical images, is utilized to identify key anatomical points on the body. These keypoints are connected to form vectors, which are then used to calculate joint angles. Furthermore, the system incorporates deep learning models to analyze motion sequences over time, enabling precise identification of gait phases such as initial contact, midstance, and toe-off by tracking changes in heel and toe positions frame by frame. KineFeet operates within a three-dimensional (3D) coordinate system (*X*, *Y*, *Z*), improving the accuracy of joint positioning and allowing for a more detailed analysis of lower limb kinematics compared to traditional two-dimensional approaches. To enhance the accuracy of angle measurement, physical reference markers (e.g., small buttons or reflective tags) may be used in combination with AI-based keypoint detection. For instance, the medial longitudinal arch (MLA) angle is calculated based on three anatomical landmarks: the medial malleolus (MM), the navicular (NV), and the head of the first metatarsal (HM), with the angle at the navicular determined using vector geometry.

$$\vec{v_{1}}=\vec{M}-\vec{N}V$$

$$\vec{v_{2}}=\vec{H}M-\vec{N}V$$

$$\cos\;\theta =\frac{\vec{v_{1}}\cdot \vec{v_{2}}}{\|\vec{v_{1}}\|\;\|\vec{v_{2}}\|}$$

$$\theta =\cos^{-1}\;\left(\frac{\vec{v_{1}}\cdot \vec{v_{2}}}{\|\vec{v_{1}}\|\;\|\vec{v_{2}}\|}\right)$$

To ensure consistent timing for gait event detection and joint angle calculation throughout the gait cycle, all video recordings were made at a rate of 30 frames per second (fps).

#### 2.1.3 Foot functional consideration underlying system development

Foot motion and muscle engagement are closely tied to three sequential functions during gait: absorbing impact, maintaining stability during weight bearing, and facilitating forward progression (13). These biomechanical roles begin at initial contact, where the heel first strikes the ground, and continue through the loading response, when body weight shifts onto the forefoot. During this early stance period, ground reaction forces induce eversion at the subtalar joint, leading to foot pronation. This movement unlocks the midtarsal joint, increasing flexibility in the tarsal region. Biomechanically, this results in flattening of the medial longitudinal arch, allowing for more uniform distribution of pressure across the foot. As the gait cycle progresses from midstance to terminal stance, the subtalar joint gradually moves into inversion, which promotes supination, a combination of plantarflexion, inversion, and adduction. Supination results in midtarsal joint locking, stiffening the tarsal structure and reelevating the medial longitudinal arch, which is essential for effective force transmission during push-off (14). Restrictions in motion at these joints may impair this natural mechanism, potentially contributing to arch instability and gait abnormalities over time. To evaluate foot kinematics that facilitate shock absorption and weightbearing stability, Kinefeet measures the subtalar angle and the Medial Longitudinal Arch angle.

##### 2.1.3.1 Subtalar angle

**Figure 5:**
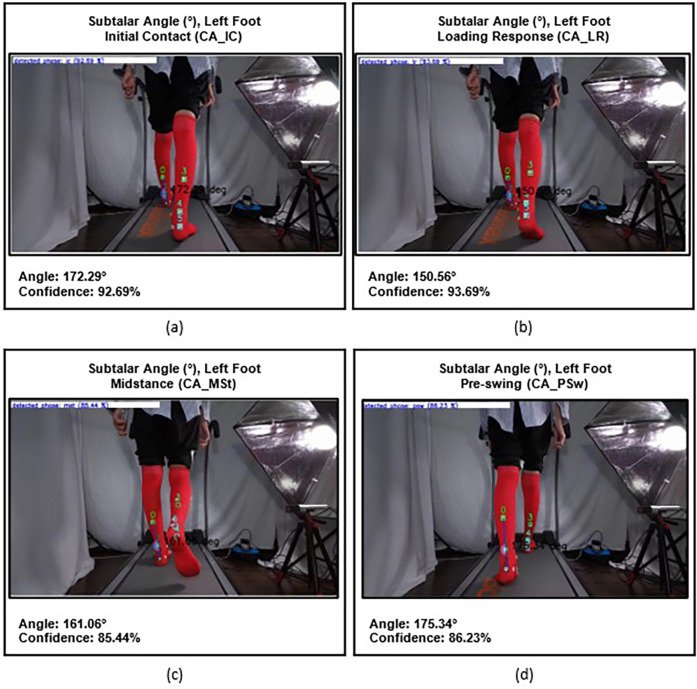
Subtalar angles of the left foot at different gait phases: **(a)** initial contact (CA_IC), **(b)** loading response (CA_LR), **(c)** midstance (CA_MSt), and **(d)** onset of pre-swing (CA_PSw).

The subtalar angle is formed by the line between the posterior calcaneus and the posterior ankle joint line, and the line between the midpoint of the tendon muscle junction and the posterior ankle joint line. The measurement was taken from the initial contact point until pre-swing, except for the terminal stance phase, due to the difficulty in accurately identifying the terminal stance position from a posterior viewpoint of the ankle (Figure 5).

##### 2.1.3.2 Medial longitudinal arch angle

**Figure 6:**
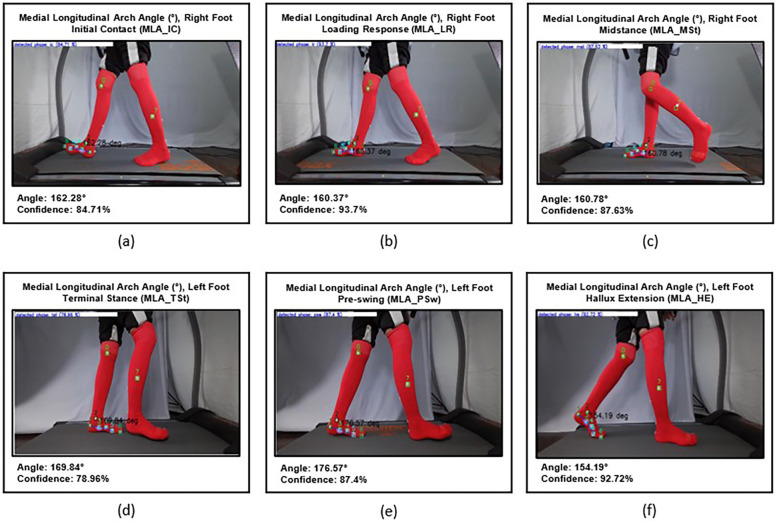
**(a)** MLA angle on initial stance phase (MLA_IC) of the right foot, **(b)** mid- loading response (MLA_LR), **(c)** midstance phase (MLA_MSt), **(d)** terminal stance phase (MLA_TSt) of the left foot, **(e)** pre-swing phase (MLA_PSw), and **(f)** maximal hallux extension (MLA_HE).

The Medial Longitudinal Arch Angle is formed by the line between the head of metatarsal 1 and the tuberosity of the navicular and the line between the tuberosity of the navicular and the posteromedial calcaneus. It was measured to capture changes in medial longitudinal arch height during the stance phase (Figure 6).

Progression during the gait cycle refers to the foot's role in effectively advancing the body forward. This is accomplished through three coordinated mechanisms known as foot rockers. First, the heel rocker is activated at the initial stance phase when the heel strikes the ground, facilitating forward rotation of the tibia while absorbing impact. Next, during mid-stance, the ankle rocker enables the tibia to progress over the planted foot through movement at the ankle joint. Finally, in the terminal stance phase, the forefoot rocker supports push-off as the heel lifts and body weight transitions to the toes. These three rockers work in tandem to ensure smooth, stable, and energy-efficient weight transfer during walking, aiding in shock absorption and minimizing energy loss (13).

**Figure 7:**
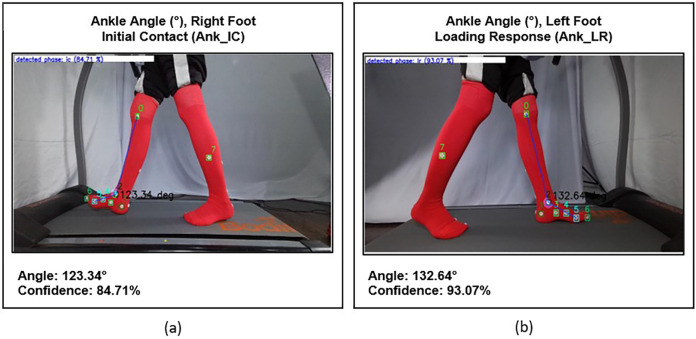
**(a)** Ankle initial contact (ANK_IC) angle of the right foot, and **(b)** ankle mid- loading response (ANK_LR) angle of the left foot.

KineFeet measures the ankle angle (Ank) to evaluate the first rocker, where foot movement occurs relative to the cruris at the talocrural joint, with the heel serving as the fulcrum. The Ankle Angle is formed by the line between the head of metatarsal 1 and the posteromedial calcaneus and the line between the medial knee joint line and the medial malleolus. It was measured at two distinct points: initial contact and mid-loading response (labeled as Ank_LR Angle). Ank_LR angles are determined when the sole is in contact with the surface and is fully supported (foot flat) (Figure 7).

**Figure 8:**
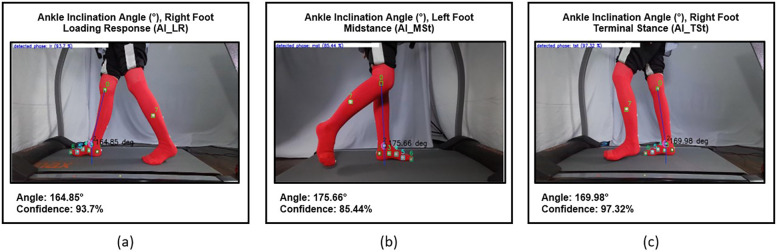
**(a)** Ankle inclination angle during mid-loading response phase (AI_LR) of the right foot, **(b)** tibia posterior inclination angle during mid-stance phase (AI_MSt) of the left foot, and **(c)** tibia anterior inclination angle during terminal stance phase (AI_TSt) of the right foot.

The ankle inclination (AI) angle is measured to evaluate the second rocker, which refers to the movement of the tibia with the talocrural joint acting as the fulcrum. This movement involves transitioning from posterior tilting in the middle of the loading response to anterior tilting at the end of midstance. The Tibia Inclination Angle is formed by a vertical line that passes through the medial malleolus and a line between the medial knee joint line and the medial malleolus. It measured during the mid-loading response, when the tibia is in a posterior inclination position, until the beginning of terminal stance, when it reaches maximum anterior inclination, just before the heel off (Figure 8).

**Figure 9:**
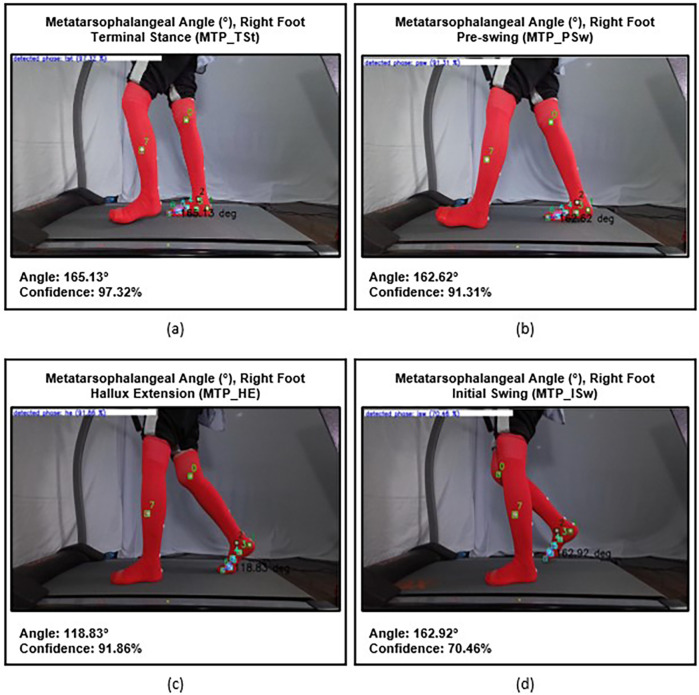
Metatarsophalangeal (MTP) angles of the right foot: **(a)** at the beginning of terminal stance phase (MTP_TSt), **(b)** during pre-swing phase (MTP_PSw), **(c)** prior to toe-off (MTP_HE), and **(d)** at the onset of initial swing (MTP_ISw).

Finally, the metatarsophalangeal 1 (MTP) angle is measured to evaluate the third rocker, which is the hallux extension movement at the MTP 1 joint. The 1st Metatarsophalangeal Angle is formed by the line between the midpoint of metatarsal 1 and the head of metatarsal 1, and the line between the head of metatarsal 1 and the medial head of proximal phalanx. It measured from the beginning of terminal stance, until it reaches its maximum of hallux extension, just before toe-off (Figure 9).

### 2.2 Step 2: software testing

Evaluating a machine learning model's performance is an important step in the development process. In this study, we use a classification model to categorize the data. Performance is assessed using two main metrics: the confusion matrix and the *F*1 score. The confusion matrix shows true positives, true negatives, false positives, and false negatives, providing details of the prediction results. The *F*1 score is the harmonic mean of precision and recall, balancing false positives and false negatives into a single metric (15–17).

#### 2.2.1 Stance subphase detection accuracy

**Figure 10:**
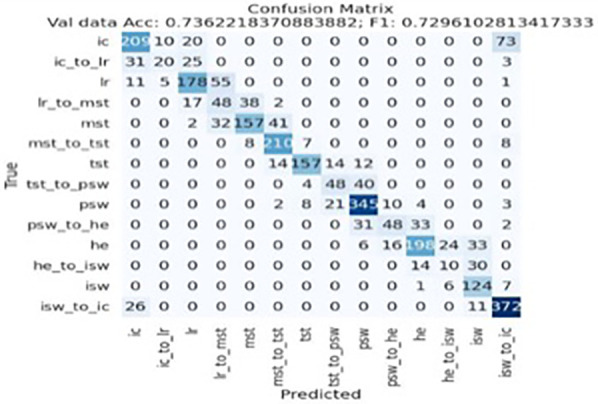
KineFeet's confusion matrix. IC, initial contact; IC_to_LR, initial contact to loading response; LR, loading response; LR_to_MSt, loading response to midstance; MSt, midstance; MSt_to_TSt, midstance to terminal stance; TSt, terminal stance; TSt_to_PSw, terminal stance to pre-swing; PSw, pre-swing; PSw_to_HE, pre-swing to hallux extension; HE, hallux extension; HE_to_ISw, hal- lux extension to initial swing; ISw, initial swing; ISw_to_IC, initial swing to initial contact.

The efficacy of Kinefeet in identifying gait subphases was assessed using a classification model that organizes data into predefined categories. The confusion matrix presented in Figure 10 provides a detailed evaluation of the multi-class classification performance across 14 discrete subphases in the validation dataset. The overall accuracy was 73.6%, with a macro-averaged *F*1-score of 0.730, suggesting moderate generalizability of the model across categories. However, class-wise analysis indicates several critical misclassification patterns that require attention (Figure 10).

To facilitate interpretation of the confusion matrix, the 14 subphases are abbreviated as follows: IC (Initial Contact)—the moment when the foot first contacts the ground, typically with the heel; IC_to_LR (Initial Contact to Loading Response)—the transition phase from heel strike to full plantar contact (metatarsal heads touching the ground); LR (Loading Response)—when the entire foot is flat on the floor, just before the tibia begins to incline anteriorly; LR_to_MSt (Loading Response to Midstance)—the transition from full-foot contact to contralateral toe-off; MSt (Midstance)—the point when the contralateral foot lifts off the ground, marking the start of single-limb support; MSt_to_TSt (Midstance to Terminal Stance)—the transition from midstance to when the tibia reaches a vertical position; TSt (Terminal Stance)—when the contralateral limb passes the stance limb and the tibia of the stance limb is upright; TSt_to_PSw (Terminal Stance to Pre-Swing)—the phase from vertical tibial alignment to initial ground contact by the contralateral foot; PSw (Pre-Swing)—beginning with contralateral contact and indicating the initiation of load transfer off the stance limb; PSw_to_HE (Pre-Swing to Hallux Extension)—transition from contralateral contact to maximal dorsiflexion of the hallux; HE (Hallux Extension)—the moment of peak hallux extension just before the metatarsal heads lift off; HE_to_ISw (Hallux Extension to Initial Swing)—the interval from maximal hallux extension to the first liftoff of the hallux; ISw (Initial Swing)—when the hallux leaves the ground, initiating the swing phase; and ISw_to_IC (Initial Swing to Initial Contact)—the transition from toe-off to the subsequent heel strike.

##### 2.2.1.1 Correct classification

The model exhibited strong performance in identifying the terminal states PSw (*n* = 345), HE (*n* = 198), and ISw_to_IC (*n* = 372), all of which showed high diagonal dominance. This indicates that these states possess distinctive features that the model successfully learned to differentiate. For instance, ISw_to_IC had minimal confusion with adjacent transitions, underscoring the separability of this class in the feature space.

##### 2.2.1.2 Misclassification pattern

**Figure 11:**
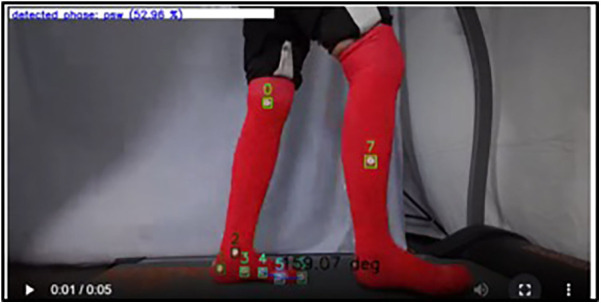
Misclassification example: TSt_to_PSw (terminal stance to pre-swing) was incorrectly classified by the model as PSw (pre-swing).

Significant class confusion was observed across gait subphases, particularly between LR, LR_to_MSt, and MSt, as well as between TSt, TSt_to_PSw, and PSw_to_HE. For instance, LR was frequently misclassified as LR_to_MSt (*n* = 48) and MSt (*n* = 55), and vice versa, indicating a lack of discriminability in these temporally adjacent subphases. Similarly, TSt_to_PSw was often confused with TSt (*n* = 48) and PSw (*n* = 40), suggesting that the model struggles to capture the nuances in state transitions, potentially due to feature overlap or temporal ambiguity (Figure 11).

Notably, IC was misclassified as ISw_to_IC in 73 instances, the largest off-diagonal error in the matrix. This misclassification likely reflects a high degree of feature similarity between baseline and transition-to-baseline states, indicating the need for improving feature engineering or temporal modeling refinements (e.g., incorporating sequential data via LSTMs or temporal attention mechanisms).

#### 2.2.2 Accuracy angle formation

**Figure 12:**
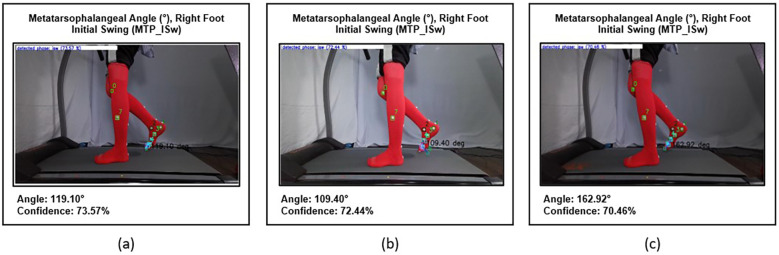
Accuracy of angle formation. **(a,b)** Show incorrect detection of markers resulting in inaccurate angle formation, while **(c)** illustrates correct marker detection producing accurate angle measurement.

Another important factor is the angle formed by two intersecting lines with markers as endpoints. This task is challenging, as the swinging contralateral limb often obscures the markers. At times, the application fails to recognize the markers as endpoints, resulting in an incorrect angle. Additionally, a consistent mistake has been observed in calculating the subtalar angle. While the angle created is correct through the chosen marker, the predicted angle size is significantly different from the actual value (Figure 12).

### 2.3 Step 3: usability testing

Usability testing is a methodological approach to evaluate the functionality of applications or systems. It involves structured user scenarios to assess the ease and efficiency with which users can navigate and interact with the system. The tester will observe the user while they utilize the application to complete the scenario task that has been assigned to the user (18).

#### 2.3.1 Methods

Usability testing of the KineFeet prototype was conducted using a user-based testing system to measure effectiveness, efficiency, and user satisfaction. The respondents were given 30 tasks, ranging from tool preparation and patient preparation to recording and downloading results. The effectiveness of an application is typically assessed by how successfully users achieve their objectives. This can be measured by the number of tasks completed, whether fully or partially, along with the frequency of errors encountered during task performance and the average time taken to complete these tasks. Time-Based Efficiency (TBE) refers to the speed at which users interact with the application while completing tasks (19). Participants were also asked to complete the System Usability Scale (SUS), a ten-item questionnaire providing a quantitative measure of perceived usability (see Supplementary Table S1) (20). After completing the trial, participants were asked to participate in an open-ended feedback interview.

#### 2.3.2 Participants

Five physiatrists with experience and in-depth knowledge of observational gait analysis were recruited for the usability evaluation.

#### 2.3.3 Data analysis

Quantitative data were analyzed using descriptive statistics, and SUS scores were calculated for each participant. The satisfaction aspect is assessed using the System Usability Scale. Each participant will be given tasks based on the task scenario that has been created (see Supplementary Table S2). After completing the given tasks, participants ought to fill the SUS questionnaire as a final step.

##### 2.3.3.1 Success rate

The success rate is considered acceptable if the average value reaches 78% (19). The component calculated by the success rate is the percentage of tasks that users complete correctly. Equation 1 is an equation for calculating the success rate, where *S* is the number of fully completed tasks (full successes), PS is the number of partially completed tasks (partial successes), and Total Tasks is the total number of tasks assigned.

(1) $$\mathrm{Success}\;\mathrm{Rate}=\;\frac{S+(\mathrm{PS}\;\times \;0.5)}{\mathrm{Total}\;\;\mathrm{Tasks}}\;\times \;100\text{\%}$$

##### 2.3.3.2 Time-based efficiency

Time-Based Efficiency, is considered normal when the user's TBE, divided by that of an expert, approaches a value of 1 (19). In this context, an expert is defined as someone who is more familiar with KineFeet and has used it extensively (more than 100 times), specifically the research team.

Time-based efficiency (TBE) is the speed at which users use the application when completing tasks. The component calculated by Time-Based Efficiency is the percentage of tasks that users complete correctly. Equation 2 is the equation used to calculate TBE. *n_ij_* is the result of task *i* by user *j*, where *n_ij_* = 1 if the task is successfully completed and *n_ij_* = 0 otherwise; *t_ij_* is the time taken by user *j* to complete task *i*; *N* is the total number of tasks; and *R* is the number of respondents.

(2) $$\mathrm{Time}\;\mathrm{Based}\;\mathrm{Efficiency}=\;\frac{\sum_{\;j=1}^{R}\;\sum_{i=1}^{N}\frac{n_{ij}}{t_{ij}\;}}{N\;R}$$

##### 2.3.3.3 Error rate

Error Rate, said to be reasonable if the average error rate value is 0.02 (19). Error Rate is the rate of errors made by users during testing. The component calculated by the error rate is interpreted as an inappropriate action or error made by the user when completing the task. Equation 3 is used to calculate the error rate. Where Total Defects refers to the number of errors or incorrect actions recorded during usability testing, and Total Opportunities is the total number of user interactions or steps where an error could potentially occur.

(3) $$\mathrm{Error}\;\;\mathrm{Rate}=\frac{\mathrm{Total}\;\mathrm{Defects}}{\mathrm{Total}\;\;\mathrm{Opportunities}}$$

##### 2.3.3.4 System usability scale (SUS)

System Usability Scale (SUS) scores were interpreted using the adjective rating scale proposed by Jeff Sauro. Scores between 84.1 and 100 indicate “Best Imaginable” usability. Scores ranging from 72.6 to 84.0 are considered “Excellent”, while scores from 62.7 to 72.5 reflect “Good” usability. A SUS score between 51.7 and 62.6 corresponds to an “OK” rating. Scores from 25.1 to 51.6 are interpreted as “Poor”, and scores between 0 and 25 represent “Worst Imaginable” usability (20).

## 3 Results

Participants demonstrated strong knowledge of gait cycle phases and kinematics, enabling them to effectively evaluate the performance of KineFeet in measuring foot kinematics.

### 3.1 Effectiveness

The average success rate for respondents in evaluating foot kinematics using KineFeet was 96.29%, with an error rate of just 0.074%. The average time taken to complete all assigned tasks was 10 min and 11 s.

### 3.2 Time-based efficiency (TBE)

The user's TBE when evaluating a non-neuropathic diabetic patient using KineFeet was 0.0442 tasks per second, while the expert's TBE was 0.05348 tasks per second. These metrics illustrate how much longer it takes a user to complete tasks compared to an expert. On average, users required 1.209 times longer to complete the tasks than the expert.

### 3.3 System usability scale (SUS)

The Questionnaire consists of 10 questions, each question is answered using a 5-point Likert scale (from Strongly Disagree to Strongly Agree). The questionnaire is designed to provide a comprehensive view of how users perceive usability across the application. In the context of the assessment, satisfaction has been identified as a primary metric, with an average Satisfaction Survey (SUS) value of 66.5 points. This indicates that the level of user satisfaction and acceptance of the application is deemed as adjectively good (in the range of 62.7–72.5). This outcome suggests that the use of KineFeet is practical.

### 3.4 User feedback

User feedback provided valuable insights into KineFeet's usability, particularly in terms of learnability, effectiveness, efficiency, and satisfaction (see Supplementary Table S3). Users found the language and terminology clear, though some expressed confusion about the sequence of result images and the unexplained Heel-Off (HE) phase. They recommended reordering the images to follow the correct gait phase sequence (IC–LR–MSt–TSt–PSw–HE–ISw) and including visual aids for better gait phase identification. Navigation was generally intuitive, but challenges were reported during the recording process, especially in cueing patients on the treadmill, maintaining sock-mounted markers, and setting up technical components like cameras and cables. Users suggested improvements such as better marker design and fixed camera mounts for bilateral recordings. Despite these issues, they reported high confidence (80%–100%) in completing tasks with minimal disruption.

KineFeet was also perceived as efficient and responsive, with users valuing features like automatic gait angle calculations and a clean interface. However, the need to run external applications (e.g., Azure Kinect Viewer, Kinect Manager) before launching KineFeet was seen as inconvenient. Streamlining this step and allowing direct video frame selection were recommended. Overall satisfaction was high, with users praising the system's clarity, ease of use, and helpful annotations. While most expectations were met, some suggested incorporating subtalar angle analysis in future versions. These findings indicate strong clinical potential, with targeted refinements likely to enhance usability and user acceptance.

## 4 Discussion

Accurate subphase start/end detection is crucial for the correct interpretation of the data. The overall accuracy was 73.6%, with a macro-averaged *F*1-score of 0.730, suggesting moderate generalizability. Users should verify several prediction options presented by Kinefeet to ensure the accuracy of subphase detection dan angle formation.

The prediction of the subtalar angle is significantly inaccurate for several reasons. First, the subtalar movement is minimal and difficult to detect using the Kinefeet camera. Second, the axis of the ankle and subtalar joints is oblique, causing slight rotation when there is eversion or inversion movement in the subtalar due to midfoot movement. Third, there is inadequate data for visual computation. To enhance the precision of subtalar angle measurement, future developments of KineFeet should involve the incorporation of a posterior camera to augment its visual computation capabilities. Consequently, the subtalar angle measurement will be excluded from future assessments of KineFeet's performance and clinical application until the necessary enhancements have been implemented and subjected to further evaluation.

Kinefeet was developed using a “machine-learned model”, which refers to a model created by applying a supervised machine learning algorithm to a labeled dataset (19). Machine-learned models are trained on specific datasets, known as their training distribution. The potential issues with this model include out-of-distribution generalization and incorrect feature attribution (21). To prevent errors related to these problems, careful evaluation of machine-learned models is essential (22), using new data from the performance distribution, including samples that are likely to reveal model failures such as those with different population demographics, challenging conditions, poor quality images, or errors (21).

The accuracy of subphase and marker detection could be improved by changing the model training approach used for automated machine learning (AutoML) or continuous machine learning (CML) in the next development project. AutoML is a process that automates the repetitive and time-consuming steps involved in creating machine learning models. This framework helps data scientists, analysts, and developers build machine learning (ML) models with high scalability, efficiency, and productivity while maintaining model quality (23, 24). CML, also called continuous learning or continual learning, is an approach where machine learning models gradually gain knowledge from new data streams over time. This process happens without needing explicit retraining on a fixed dataset. Unlike traditional machine learning models, which are trained once on a static dataset and then retrained periodically, continuous learning models go through an iterative process of updating their parameters. This allows the models to adapt to new data patterns and changing conditions in real time (24).

The usability test supports KineFeet as a feasible tool for clinical foot kinematics analysis, particularly in settings where high-end motion capture systems are unavailable. The high success rate and low score rate indicate that KineFeet is an easy application to use. The TBE score, which is close to one, also indicates that KineFeet is easy to learn, so that the time taken to complete the examination protocol is not significantly different between a new user and an expert. Additionally, the SUS score of 66.5 indicates that clinicians found the system to be intuitive and functional for its intended use. From the interviews, it was found that the participants thought KineFeet had a very promising potential. Participant feedback highlighted areas for further improvement, particularly simplifying the setup process and reducing reliance on external applications such as Azure Kinect Viewer, which could make the system even more practical for routine clinical use.

Limitations of the study include a small sample size and a focus on qualitative evaluation and usability rather than accuracy or validity. Prior research has shown that a small number of participants—approximately four to five users—can reveal a significant portion of usability issues. For example, Nielsen and Molich reported that five evaluators identified about two-thirds of usability problems using heuristic evaluation (25), while Virzi demonstrated that four or five users are often sufficient to detect around 80% of usability issues using think-aloud protocols (26). Future work will therefore focus on clinical validation of measurement accuracy against gold-standard systems, test-retest reliability testing, and expanding usability assessments to larger and more diverse user groups—including clinicians, technicians, and patients—to enhance both generalizability and clinical relevance.

## 5 Conclusion

KineFeet represents a promising innovation in clinical foot kinematics assessment. Its web-based architecture and use of depth camera technology make it a practical alternative to traditional gait labs. The system demonstrated strong usability in preliminary testing and holds potential for broader clinical adoption following further development.

## Data Availability

The raw data supporting the conclusions of this article will be made available by the authors, without undue reservation.
