# Supplementary material for: Development and usability testing of a depth camera–based web application for functionally relevant foot kinematics analysis
Source: Front Med Technol. 2025 Oct 3;7:1677174. doi: 10.3389/fmedt.2025.1677174 (PMC12531154; doi:10.3389/fmedt.2025.1677174)
Supplement: Supplementary file 1 [file Table1.docx]

Supplementary Material

# Supplementary Table 1

Table 1. System Usability Scale (SUS) Questionnaire in KineFeet Usability Testing

| **No** | **Questionnaire** |
| --- | --- |
| 1 | I think that I would like to use this product frequently |
| 2 | I found the product unnecessarily complex |
| 3 | I thought the product was easy to use |
| 4 | I think that I would need the support of a technical person to be able to use this product |
| 5 | I found the various functions in the product were well integrated |
| 6 | I thought there was too much inconsistency in this product |
| 7 | I imagine that most people would learn to use this product very quickly |
| 8 | I found the product very awkward to use |
| 9 | I felt very confident using the product |
| 10 | I needed to learn a lot of things before I could get going with this product |

# Note: The questionnaire consists of 10 items rated on a 5-point Likert scale (Strongly Disagree to Strongly Agree). In this study, the average SUS score was 66.5, which falls within the good usability range (62.7–72.5).

# Supplementary Table 2

Table 2. Task Scenario in User-Based Testing of the KineFeet System

| **Task Code** | **Task** | **Details** |
| --- | --- | --- |
| T1 | Prepare equipments for KineFeet recording | Place cameras and light-source |
|  |  | Turn on the light-source |
|  |  | Make sure the camera screen display on Azure Kinect Viewer v1.4.1 is correct. |
| T2 | Prepare the patient | Ensure the patients wear shorts above knee |
|  |  | Prepare sets of red sock and leg-sleeve (if needed) |
|  |  | Place the markers on patient's foot according to guidance |
| T3 | System login and examination initiations | Enter correct website in browser |
|  |  | Log-in to the application |
|  |  | Able to start an examination in new patient by clicking *Pemeriksaan* |
|  |  | Enter and save new patient's data |
|  |  | Ensure date and patient's data which was entered before are correct |
|  |  | Open the Kinectron application |
|  |  | Return to KineFeet’s web and start the examination by clicking *Periksa* button |
| T4 | Video recording | Ensure that the camera screen is displayed on the KineFeet’s web |
|  |  | Ensure the correct recording of the foot by clicking LEFT or RIGHT in Position panel |
|  |  | Able to lead the patients and provide instructions for familiarization to walk on treadmill up to the speed of 2km/hour. |
|  |  | Able to assess that the patient is in natural and stable walking speed and position |
|  |  | Able to record - Click *Mulai merekam* |
|  |  | Record the video for 10 seconds |
|  |  | Wait for the process of saving the video is complete |
|  |  | Submit recording - Click *Submit* |
|  |  | Wait for the process of video analysis and upload of videos and images are complete |
| T5 | Evaluate the results of recording and download the report | Open the result of recording- Click *Detail Pemeriksaan* |
|  |  | Ensure the temporal parameters are listed correctly; stance phase, Gait cycle, Stance phase time percentage, forefoot contact time, forefoot contact time percentage, heel contact time, and heel contact percentage |
|  |  | Ensure the images and videos capture the markers well |
|  |  | Ensure and assess the Medial longitudinal arch (MLA) angle from each gait phase either in the form of images or videos are correct |
|  |  | Ensure and assess the Metatarsal Phalangeal 1 (MTP) angle from each gait phase either in the form of images or videos are correct |
|  |  | Ensure and assess the Ankle (ANK) angle and Talocrural Inclination (AI) from each gait phase either in the form of images or videos are correct |
|  |  | Able to download report - click *Download PDF* |

Note: This table outlines the five main task scenarios used in the usability test, from system setup and patient preparation to recording, analysis, and report generation. Participants achieved a high task completion rate, with an average success rate of 96.29%, an error rate of 0.074%, and an average completion time of 10 minutes 11 seconds.

# Supplementary Table 3

Table 3. Qualitative Assessment Results of KineFeet Application Based on User Feedback

| **Aspect** | **Question** | **Feedback** | **Recommendation** |
| --- | --- | --- | --- |
| Learnability | Was the language or terminology easy to understand? | Users agree that the language and terminology use in KineFeet are easy to understand |  |
|  | What aspect in KineFeet felt unclear or confusing? | A user thought that the list of result pictures are a bit confusing since it isn't in order.  Some user realise that Gait Phases of HE doesn't have an explanation | Fix the order of the results according to gait phases (IC-LR-MSt-TSt-PSw-HE_ISw).  Give explanation on what HE gait phase means |
|  | What additional information would have made this easier for future user of KineFeet? | A user suggests to make image display of gait phase guidance in KineFeet thus users can visualize and chose correctly which picture represents each gait phase best. |  |
| Effectiveness | How would you describe the navigation experience while using KineFeet? | Users feel that there is no obstacle in navigating KineFeet application |  |
|  | What part in recording process of KineFeet was most challenging, and why? | Some users felt that the challenging part in cueing patient to walk on treadmill, and the part where the button markers are easy to fall from socks.  Some users agreed that the settings and switch the cameras, cables, and making sure that the camera is connected to the computer are quite challenging. | A user suggest to improve the marker therefore easier to use and uneasy to fall or misplaced  Make camera mounted on fix structures such as wall or hanging from the ceiling and program the ability to record both legs (right and left) on the same time |
|  | Were there any points where you felt stuck? Can you explain what happened? | Users claim to have no hindrance while using KineFeet. |  |
|  | How confident did you feel while completing recording process of KineFeet? | Users feel 80-100% confidence in completing KineFeet tasks. |  |
| Efficiency | How did you feel about the overall speed and responsiveness? | Users agree that overall speed and responsiveness are good. |  |
|  | Did anything about the layout or design distract you? | Users found no distraction. |  |
|  | How does KineFeet compare to similar application or software you’ve used? | Compare to software users usually use, KineFeet is easier and efficience in time wise since the angle in each gait phases are automatically calculated. |  |
|  | What would it be if you could change one thing about KineFeet for future advancement? | Some users hope to simplify the steps to use KineFeet in terms of users don't have to open multiple applications (Azure Kinect Viewer to see whether the camera placements are align, Kinect Manager to confirm cameras are connecting to the computer) before utilizing KineFeet.  Since not all angle in gait phases seen clearly in picture user are confuse on what to do after taking the correct angle in videos. | Minimalize page-switching.  If all five pictures are not representing correct angle in a gait phase and users have to choose from the videos, make the chose frame in video able to appear on results page. |
| Satisfaction | What were your initial impressions when you saw KineFeet interface? | The interface is clear and easy to navigate. |  |
|  | What KineFeet feature do you like the most, and why? | Users like that the picture automatically shows the angle and its confidence level, therefore the user knows which result has the highest confidence level and what angle to choose amongs the 5 chosen pictures.  Users like that there are explanation beside the picture about the gait phase. |  |
|  | Do you feel KineFeet meets your expectations? Why or why not? | Almost meet the expectation, however users hope subtalar angle can be use in the future. | Improve the ability to analyze subtalar angle. |

Note: This table summarizes qualitative feedback from participants across four aspects of usability—learnability, effectiveness, efficiency, and satisfaction. Users reported clear terminology, intuitive navigation, and high confidence (80–100%) in task completion.
